# Identification of two novel genes *SLC15A2* and *SLCO1B3* associated with maintenance dose variability of warfarin in a Chinese population

**DOI:** 10.1038/s41598-017-17731-1

**Published:** 2017-12-12

**Authors:** Liang-Liang Cai, Wen-Qing Huang, Zhi-Ying Su, Hui-Ming Ye, Lian-Sheng Wang, Yuan Wu, Zhong-Ying Zhang, Wei Zhang, Chi-Meng Tzeng

**Affiliations:** 1 Translational Medicine Research Center, School of Pharmaceutical Sciences, Xiamen University, Xiamen, Fujian Sheng, China; 2 Clinical Research Laboratory, Xiamen’s Maternal and Child Health Hospital, Teaching Hospital of Xiamen University, Xiamen, Fujian Sheng, China; 3 Department of Clinical Pharmacology, Xiangya Hospital, Institute of Clinical Pharmacology, Central South University, Changsha, Hunan Sheng, China; 4 Department of cardiac surgery, Xiamen Cardiovascular Hospital, School of Medicine, Xiamen University, Xiamen, Fujian Sheng, China; 5 Department of Clinical laboratory, Zhongshan Hospital, Xiamen University, Xiamen, Fujian Sheng, China

## Abstract

Warfarin is a commonly prescribed and effective oral anticoagulant. Genetic polymorphisms associated with warfarin metabolism and sensitivity have been implicated in the wide inter-individual dose variation that is observed. Several algorithms integrating patients’ clinical characteristics and genetic polymorphism information have been explored to predict warfarin dose. However, most of these algorithms could explain only over half of the variation in a warfarin maintenance dose, suggesting that additional genetic factors may exist and need to be identified. Here, a drug absorption, distribution, metabolism and excretion (ADME) Core Panel Kit-based pharmacogenetic study was performed to screen for warfarin dose-associated SNP sites in Han-Chinese population patients taking warfarin therapy, and the screen was followed by pyrosequencing-based validation. Finally, we confirmed that the common variant rs9923231 in VKORC1 and two novel genes, SLC15A2 (rs1143671 and rs1143672) and SLCO1B3 (rs4149117 and rs7311358), are associated with the warfarin maintenance dose. As has been shown for those carriers with the variant rs9923231 in VKORC1, it was suggested that those subjects with homozygous minor alleles in those four SNPs should take a lower warfarin dose than those carrying the wild type alleles. Together with the established predictor rs9923231 in VKORC1, those four novel variants on SLC15A2 and SLCO1B3 should be considered as useful biomarkers for warfarin dose adjustment in clinical practice in Han-Chinese populations.

## Introduction

Warfarin is a commonly prescribed and effective oral anticoagulant for treating and preventing thrombotic events in patients with prosthetic heart valves, deep venous thromboembolism, pulmonary embolism and atrial fibrillation. It is highly effective but has a narrow therapeutic window and wide inter-individual variation in the daily dose required, and these factors increase the risk for haemorrhagic complications and pose challenges for achieving adequate anticoagulation^1–4^. Therefore, careful monitoring is needed together with accurate and frequent dose adjustment for the prevention of adverse drug reactions (ADRs) related to the use of warfarin. Inter-individual variability in warfarin dose requirement is a major clinical problem and may occur due to the influence of many factors, such as age, weight, height, genetic variants, disease and drug interactions^5,6^. Genetic variations in those genes associated with the pharmacodynamics and pharmacokinetics of warfarin are considered to be determinants of the dose requirement^7^.

To date, several genome-wide association studies (GWAS) of warfarin dosing requirements have been performed in various ethnic populations, such as White/Caucasian, Brazilian and Swedish, African American and Japanese populations^8–12^. Together with some candidate gene studies^13,14^, they have revealed many genetic polymorphisms with large effects on warfarin dose, including *VKORC1, CYP2C9*, and *CYP4F* ^8–12^. Furthermore, since the beginning of 2005, many algorithms for predicting the appropriate dose of warfarin using certain genetic polymorphisms, such as *VKORC1*-1639 G > A (rs9923231), CYP2C9*2 (rs1799853), and CYP2C9*3 (rs1057910), in addition to clinical factors have been developed for use in warfarin dose prediction. However, until now, most of these algorithms could only explain over half of the variation in warfarin maintenance dose^6,15–18^. In a recent study, the performance of 8 warfarin pharmacogenetic algorithms was compared in Chinese patients, and no eligible algorithm performed the best for all dosing ranges under low intensity warfarin anticoagulation^19^. To elevate the performance of warfarin pharmacogenetic algorithms, it is important and necessary to screen for additional genetic factors.

As previously mentioned, an additional benefit to some pharmacogenomics studies is the ability to study of the mechanism of the drug’s action. Regarding this point, more targets were involved in the initial design of the molecular study. For example, several commercial products have been designed as genotyping arrays specifically focused on those genes associated with drug transport and drug metabolism, such as the Affymetrix ADME chip and the Illumina ADME chip. Platforms such as those allow for more targeted evaluation of genes known to be related to the drug and (or) phenotype of interest. The Illumina VeraCode ADME Core Panel interrogates the key gene biomarkers associated with ADME using allele-specific extension and ligation followed by PCR with fluorescently labelled primers. This panel enables more comprehensive coverage of the most biologically relevant biomarkers spanning complex regions of the genome and is more efficient, with results in as fast as one day. This study aimed to ascertain the influence of 184 key genetic variants of 34 key ADME genes on the required warfarin maintenance dose in Han-Chinese patients.

## Results

### Patient characteristics

The first screening cohort of 156 patients was recruited from the department of clinical laboratory, Zhongshan hospital, Xiamen University, from 2012 to 2014. Among those patients, 36 (23.1%) were included in the high warfarin dose group, which had therapeutic warfarin dose requirements of more than 4 mg/day, and 32 (20.5%) were included in the low warfarin dose group, which had therapeutic warfarin dose requirements of less than 2 mg/day. The remaining 88 patients (56.4%) were classified into the medium warfarin dose group, which had therapeutic warfarin dose requirements between 2 and 4 mg/day. Those three groups of patients consented to take part in the ADME microarray screening of risk variants associated with warfarin maintenance dose. Among those patients in the “screening cohort”, 144 patients yielded sequences with good sequencing quality, while the other 12 patients with poor DNA quality failed in the ADME microarray screening. Together with a second cohort of 57 patients recruited from this hospital in 2015, those 144 patients were included in “replication cohort” which was further used in the pyrosequencing validation of risk SNPs identified from the ADME microarray screening study. Therefore, a total of 201 patients were finally included in the study. The baseline demographics and clinical characteristics of those patients are shown in Table 1. The average age was 52.8 ± 11.8 years, and 85 patients were male (42.3%). Additionally, the average height and weight were 162.6 ± 7.3 cm and 60.5 ± 10.9 kg, respectively. The mean warfarin dose was 3.14 ± 1.41 mg/day, ranging from 0.56 mg/day to 9.0 mg/day; 44 patients (21.9%) had a therapeutic warfarin dose requirement of less than 2 mg/day, 112 patients (55.7%) required between 2 and 4 mg/day and 45 patients (22.4%) required more than 4 mg/day. Among the above-mentioned clinical covariates, warfarin dose was not significantly influenced by gender (χ^2^ = 1.973, p = 0.163), height (χ^2^ = 0.864, p = 0.350) or weight (χ^2^ = 0.752, p = 0.403) but was influenced by age (χ^2^ = 10.849, p = 0.001). The indications for warfarin therapy were heart valve replacement (80.6%), atrial fibrillation (15.4%) and deep vein thrombosis and pulmonary embolism (4.0%).

**Table 1:** Baseline characteristics of the study population.

**Demographic characteristics**	**Patients**
**Age (years)**	52.8 ± 11.8
**Gender [n (%)]**	
Male	85 (42.3%)
Female	116 (57.7%)
**Weight (kg)**	60.5 ± 10.9
**Height (cm)**	162.6 ± 7.3
**Combination use of amiodarone [n (%)]**	5 (2.5%)
**Combination use of enzyme inhibitor [n (%)]**	18 (9.0%)
**Stable warfarin dose (mg/day)**	3.14 ± 1.41
**Indications [n (%)]**	
Atrial fibrillation	31 (15.4%)
Heart valve replacement^#^	162 (80.6%)
Deep vein thrombosis	8 (4%)

^#^Twelve patients have heart valve replacement with atrial fibrillation, 1 patient has heart valve replacement with deep vein thrombosis.

### Identification of genetic variants associated with warfarin maintenance dose

**Figure 1:**
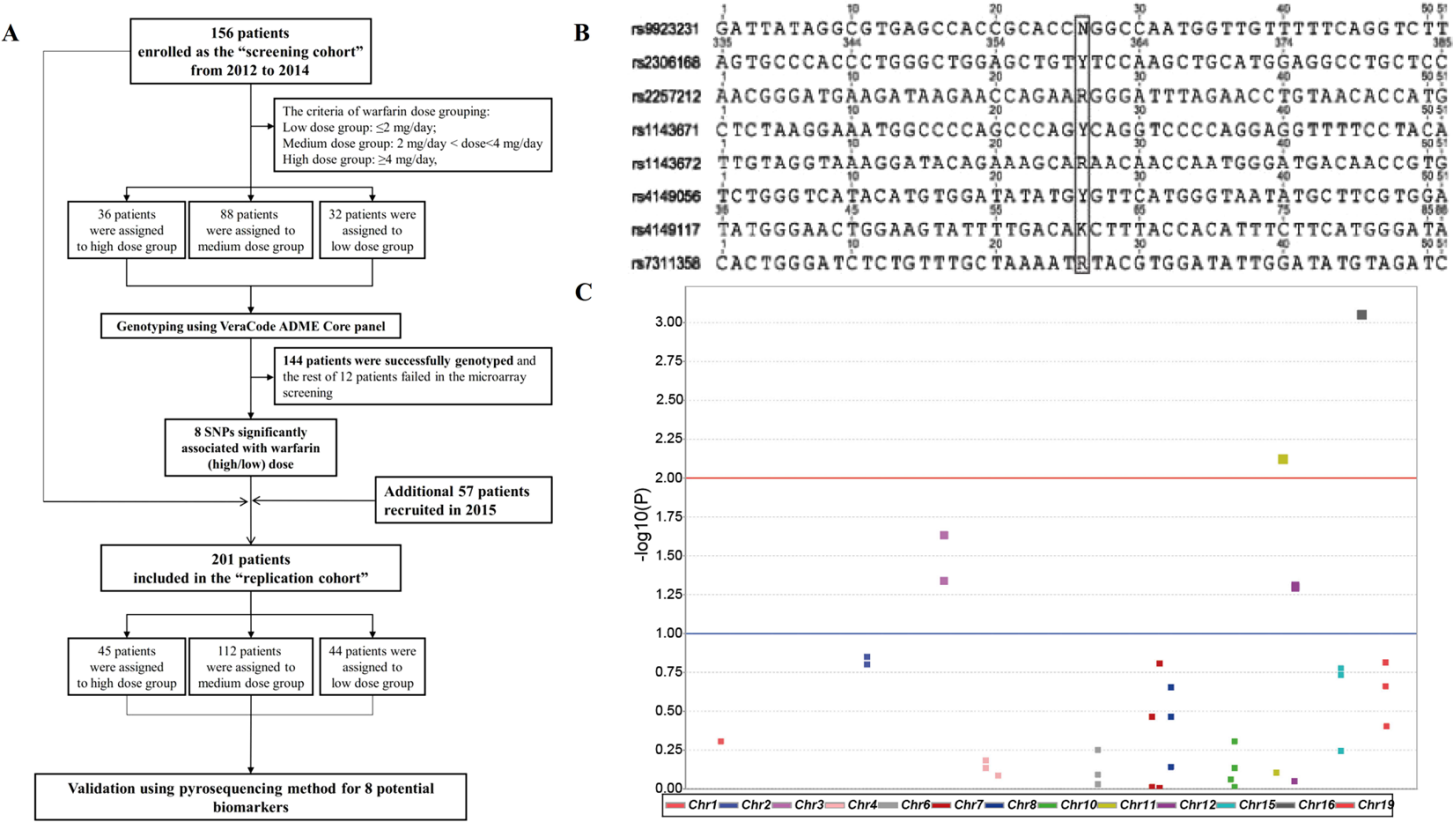
The study design and significant SNPs identified in the ADME microarray analysis. (**A**) The study design is shown as a flowchart of study enrolment and genotyping. (**B**) Eight significant SNPs were identified from a VeraCode ADME core panel-based microarray screening of warfarin pharmacogenetics in 144 enrolled patients. (**C**) All SNPs on the VeraCode ADME core panel are shown in the Manhattan plot. The X and Y axes represent the SNP genomic positions and p values, respectively. Both horizontal lines that are produced by the Haploview software indicate the threshold for significance. Among those SNPs, 8 SNPs show significance with −log_10_P > 1.3 (p < 0.05). Among those SNPs, rs9923231 in *VKORC1* and rs2306168 in *SLCO2B1* have a significance level of −log_10_P > 2.0. The other 6 SNPs (rs2257212, rs1143671 and rs1143672 in *SLC15A2*, rs4149117 and rs7311358 in *SLCO1B3*, and rs4149056 in *SLCO1B1*) possessed significance levels of −log_10_P > 1.0, and three of those SNPs in SLC15A2 (rs2257212, rs1143671 and rs1143672) with the same p value overlap, and two SNPs in SLCO1B3 (rs4149117 and rs7311358) with the same p value also overlap.

To decipher the genetic variants associated with warfarin maintenance dose in this Chinese population, we performed a large genotyping analysis of 153 candidate SNPs using the Illumina VeraCode ADME Core Panel for the 156 enrolled patients. Among those candidate variants, 9 SNPs were removed from the analysis because they showed a low call rate (<70%) during genotyping. The average call rate of the remaining 144 SNPs assayed was more than 90%. Finally, 72 SNPs across 30 genes showing a minor allele frequency greater than 1% were used to conduct the association analysis using PLINK (shown on the website: http://zzz.bwh.harvard.edu/plink/tutorial.shtml#t1)^20^ and were visualized using Haploview^21^. As shown in Table 2, those 30 genes and 72 SNPs were included in the PharmaADME panel of ADME genes (see http://pharmaadme.org/). In the association analysis, there were 8 high risk SNPs with significance values of −log_10_p > 1.0 (Fig. 1B–C). Those risk variants included rs9923231 in *VKORC1*, rs2257212, rs1143671 and rs1143672 in *SLC15A2*, rs4149117 and rs7311358 in *SLCO1B3*, rs4149056 in *SLCO1B1* and rs2306168 in *SLCO2B1*, and they were significantly associated with the warfarin maintenance dose (Table 3). Among those risk SNPs, rs9923231 in *VKORC1* stood out dramatically in the association analysis and showed a strong association with warfarin therapeutic dose. Therefore, the above 8 SNPs may represent the biomarkers for the warfarin maintenance dose in Chinese populations.

**Table 2:** The core PharmaADME genes containing SNPs with minor allele frequencies greater than 1% in the southern Han-Chinese population.

Phase I enzymes		Phase II enzymes		Transporters and Other Genes	
Gene	SNP count	Gene	SNP count	Gene	SNP count
CYP2C9	2	SULT1A1	1	SLC22A2	1
CYP2C8	1	UGT2B15	1	SLCO1B3	2
CYP2B6	2	UGT1A1	3	SLCO2B1	1
CYP2D6	3	UGT2B7	1	SLC15A2	4
CYP3A4	1	DPYD	4	SLC22A1	3
CYP3A5	2	GSTM1	1	SLCO1B1	2
CYP1A1	1	GSTP1	1	ABCB1	6
CYP1A2	3	NAT1	5	ABCC2	6
CYP2A6	3	NAT2	6	ABCG2	2
CYP2C19	2	TPMT	1	VKORC1*	1

^*^VKORC1 is the therapeutic target of warfarin and is associated with sensitivity to the drug.

**Table 3:** Eight significant SNP sites identified from the VeraCode ADME Core Panel.

CHR	SNP	POSITION	A1	F_A	F_U	A2	CHISQ	P	OR
16	rs9923231	31107689	G	0.325	0.04545	A	11.16	0.0008347	10.11
11	rs2306168	74907582	T	0.425	0.1591	C	7.259	0.007053	3.907
3	rs2257212	121643804	C	0.475	0.2391	T	5.24	0.02207	2.879
3	rs1143671	121647286	C	0.475	0.2391	T	5.24	0.02207	2.879
3	rs1143672	121648168	G	0.475	0.2391	A	5.24	0.02207	2.879
12	rs4149056	21331549	C	0.175	0.04348	T	3.95	0.04688	4.667
12	rs4149117	21011480	T	0.275	0.1087	G	3.908	0.04807	3.11
12	rs7311358	21015760	G	0.275	0.1087	A	3.908	0.04807	3.11

Note: CHR: Chromosome; SNP: SNP ID; BP: Physical position (base-pair); A1: Minor allele name (based on whole sample); F_A: Frequency of this allele in high dose group; F_U: Frequency of this allele in low dose group; A2: Major allele name; CHISQ: Basic allelic chi-square test (1df); P: Asymptotic p-value for this test; OR: Estimated odds ratio (for A1, i.e., A2 is the reference).

### The effect of VKORC1, SLC15A2, SLCO1B1, SLCO1B3, and SLCO2B1 polymorphisms on warfarin maintenance dose

To verify the ADME microarray data, we performed a genotyping analysis of the above 8 risk variants for warfarin maintenance dose in 201 subjects including the screening cohort of 144 patients using pyrosequencing technology. The pyrosequencing methods were developed to identify each SNP among the 8 SNPs in Table 4. As shown in Table 5, except for the three variants rs2306168 in *SLCO2B1*, rs4149056 in *SLCO1B1* and rs2257212 in *SLC15A2*, the other 5 SNPs were shown to be significantly associated with the warfarin maintenance dose. Among those significant variants, 3 SNPs, including rs9923231 in *VKORC1*, rs1143671 in *SLC15A2* and rs4149117 in *SLCO1B3*, were significantly associated with warfarin maintenance dose both in the overall genetic model and in a recessive genetic model. *SLC15A2* rs1143672 and *SLCO1B3* rs7311358 showed a significant association only in the recessive genetic model and the overall genetic model, respectively. In addition, the association analysis by a multiplicative model revealed that the minor alleles of those 5 risk polymorphisms were significantly related with warfarin maintenance dose. However, except for rs9923231 in *VKORC1*, the significant differences for the other 4 SNPs among the three groups of subjects with different warfarin maintenance doses was lost after the Bonferroni correction (Table 5). Moreover, in accordance with the results after adjusting for multiple testing, the significant associations of those 4 SNPs (rs1143671 and rs1143672 in *SLC15A2*, rs4149117 and rs7311358 in *SLCO1B3*) with warfarin dose were also lost after adjustment for age (Table 5). Surprisingly, one variant, rs4149056 in *SLCO1B1*, did not show a significant association with warfarin dose after Bonferroni corrections for multiple testing but was significant after adjustment for the clinical factor, age (p < 0.05), suggesting a potential importance of rs4149056 in warfarin dose adjustment (Table 5). Therefore, the significance of those SNPs, including rs9923231 in *VKORC1*, rs1143671 and rs1143672 in *SLC15A2*, rs4149117 and rs7311358 in *SLCO1B3*, and rs4149056 in *SLCO1B1* in warfarin dose prediction had to be assessed using other warfarin dosing algorithms.

**Table 4:** PCR and pyrosequencing primers for the detection of 8 significant SNPs.

**SNP**		**Sequence**
rs9923231	Forward	5′-GGGAAGTCAAGCAAGAGAAGACCT-3′
	Reverse	5′-bio-GCCTCCAGGGTTCAAGTGG-3′
	Sequencing	5′-CCTGAAAAACAACCATTG-3′
rs2306168	Forward	5′-ATACTTGCCCACCTTCATTGC-3′
	Reverse	5′-bio-CAGGGTTAAAGCCGTCCAA-3′
	Sequencing	5′-CTGGGCTGGAGCTGT-3′
rs2257212	Forward	5′-TGCAGTGCTCACACTCATGATAG-3′
	Reverse	5′-bio-TCAAACAACGGGATGAAGATAA-3′
	Sequencing	5′-TGGTGTTACAGGTTCTAAAT-3′
rs1143671	Forward	5′-TGACATGTGTTCTTTGCTCTAAGG-3′
	Reverse	5′-bio-TTGTTTTCATTTCCCACCACTG-3′
	Sequencing	5′-AAATGGCCCCAGCCC-3′
rs1143672	Forward	5′-CGTGAAGATGGGAACAGTATCTC-3′
	Reverse	5′-bio-GAATGCAGCCCCAAAGATTA-3′
	Sequencing	5′-GGTAAAGGATACAGAAAGC-3′
rs4149056	Forward	5′-bio-AAGGAATCTGGGTCATACATGTGG-3′
	Reverse	5′-GCGAAATCATCAATGTAAGAAAGC-3′
	Sequencing	5′-AAGCATATTACCCATGAAC-3′
rs4149117	Forward	5′-bio-GTTGTCTCCTTATGGGAACTGG-3′
	Reverse	5′-GCTGATAATAAATGGCTCAGAGCT-3′
	Sequencing	5′-CCCATGAAGAAATGTGGTA-3′
rs7311358	Forward	5′-AGTCATTGGCTTTGCACTGG-3′
	Reverse	5′-bio-GAAGAATGGTGTCCTGCACTTAA-3′
	Sequencing	5′-GGATCTCTGTTTGCTAAAAT-3′

Note: bio: biotinylated at the 5′end of the primer; SNP: single nucleotide polymorphism.

**Table 5:** Statistical analysis of genotypic frequencies among the high, medium and low dose groups.

**Gene**	**SNPs**	**Genotype**	**High(n = 45)**		**Medium(n = 112)**		**Low(n = 44)**		**χ** ^**2**^ **value**	**P value**	**P**_**c**_	**P**_**adj**_
			**No**.	**%**	**No**.	**%**	**No**.	**%**				
***VKORC1***	**rs9923231 (−1639G > A)**	GG	3	6.7	2	1.9	0	0	35.623	**3.46E-7** ^*^	**2.77E-6** ^*^	**1.21E-6** ^*^
		AG	22	48.9	13	12.5	3	7.0				
		AA	20	44.4	89	85.6	40	93.0				
		AG + AA (D)	42	93.3	102	98.1	43	1	4.539	0.103	0.824	0.112
		AG + GG (R)	25	55.6	15	14.4	3	7.0	38.155	**5.18E-9** ^*^	**4.14E-8** ^*^	**8.42E-7** ^*^
		G	28	31.1	17	8.2	3	3.5	38.447	**4.48E-9** ^*^	**3.58E-8** ^*^	—
		A	62	68.9	191	91.8	83	96.5				
***SLCO2B1***	**rs2306168 (c.1457 C > T)**	CC	22	50.0	64	58.7	29	67.4	3.845	0.427	1.000	0.376
		CT	15	34.1	28	25.7	7	16.3				
		TT	7	15.9	17	15.6	7	16.3				
		CT + TT (D)	22	50.0	45	41.3	14	32.6	2.729	0.256	1.000	0.152
		CT + CC (R)	37	84.1	92	84.4	36	83.7	0.011	0.994	1.000	0.979
		C	59	67.0	156	71.6	65	75.6	1.557	0.459	1.000	**—**
		T	29	33.0	62	28.4	21	24.4				
***SLCO1B1***	**rs4149056 (c.521 A > G)**	AA	31	68.9	83	75.5	38	86.4	6.304	0.121	0.968	**0.031** ^*^
		AG	13	28.9	27	24.5	6	13.6				
		GG	1	2.2	0	0	0	0				
		AG + GG (D)	14	31.1	27	24.5	6	13.6	3.883	0.143	1.000	0.059
		AG + AA (R)	44	97.8	110	1	44	1	2.896	0.447	1.000	0.562
		A	75	83.3	193	87.7	82	93.2	4.090	0.129	1.000	**—**
		G	15	16.7	27	12.3	6	6.8				
***SLC15A2***	**rs2257212 (c.1048 C > T)**	CC	8	17.8	16	16.0	3	7.1	4.995	0.288	1.000	0.613
		CT	19	42.2	30	30.0	15	35.7				
		TT	18	40.0	54	54.0	24	57.1				
		CT + TT (D)	37	82.2	84	84.0	39	92.9	2.413	0.299	1.000	0.889
		CC + CT (R)	27	60.0	46	46.0	18	42.9	3.166	0.205	1.000	0.232
		C	35	38.9	62	31.0	21	25.0	3.941	0.139	1.000	**—**
		T	55	61.1	138	69.0	63	75.0				
***SLC15A2***	**rs1143672 (c.1526 G > A)**	GG	9	20.5	14	12.8	2	4.5	9.304	0.054	0.432	0.099
		AG	19	43.2	32	29.4	16	36.4				
		AA	16	36.4	63	57.8	26	59.1				
		AG + AA (D)	35	79.5	95	87.2	42	95.5	3.482	0.175	1.000	0.140
		GG + AG (R)	28	63.6	46	42.2	18	40.9	6.549	**0.038** ^*^	0.304	0.303
		G	37	42.0	60	27.5	20	22.7	8.969	**0.011** ^*^	0.088	**—**
		A	51	58.0	158	72.5	68	77.3				
***SLC15A2***	**rs1143671 (c.1225 C > T)**	CC	8	17.8	15	13.9	2	4.5	10.836	**0.028** ^*^	0.224	0.081
		CT	20	44.4	27	25.0	14	31.8				
		TT	17	37.8	66	61.1	28	63.6				
		CT + TT (D)	37	82.2	93	86.1	42	95.5	3.826	0.148	1.000	0.268
		CC + CT (R)	28	62.2	42	38.9	16	36.4	8.255	**0.016** ^*^	0.128	0.092
		C	36	40.0	57	26.4	18	20.5	9.152	**0.010** ^*^	0.080	**—**
		T	54	60.0	159	73.6	70	79.5				
***SLCO1B3***	**rs4149117 (c.334 A > C)**	AA	1	2.2	6	5.7	0	0	10.629	**0.031** ^*^	0.248	0.104
		AC	24	53.3	46	43.4	13	29.5				
		CC	20	44.4	54	50.9	31	70.5				
		AC + CC (D)	44	97.8	100	94.3	44	1	4.620	0.099	0.792	0.867
		AA + AC (R)	25	55.6	52	49.1	13	29.5	6.844	**0.033** ^*^	0.264	0.069
		A	26	28.9	58	27.4	13	14.8	6.282	**0.043** ^*^	0.344	**—**
		C	64	71.1	154	72.6	75	85.2				
***SLCO1B3***	**rs7311358 (c.699 G > A)**	GG	2	4.4	8	7.3	0	0	9.514	**0.049** ^*^	0.392	0.141
		AG	21	46.7	45	40.9	13	29.5				
		AA	22	48.9	57	51.8	31	70.5				
		AG + AA (D)	43	95.6	102	92.7	44	1	5.599	0.061	0.488	0.662
		GG + AG (R)	23	51.1	53	48.2	13	29.5	5.375	0.068	0.544	0.124
		G	25	27.8	61	27.7	13	14.8	6.170	**0.046** ^*^	0.368	**—**
		A	65	72.2	159	72.3	75	85.2				

Note: CI = confidence interval, D = dominant model, OR = odds ratios, Padj = Padjusted, which was adjusted for age. Pc = P corrected, which was corrected for multiple testing using the Bonferroni method, R = recessive model, SNPs = single nucleotide polymorphisms. ^∗^Significant association with p value < 0.05.

**Figure 2:**
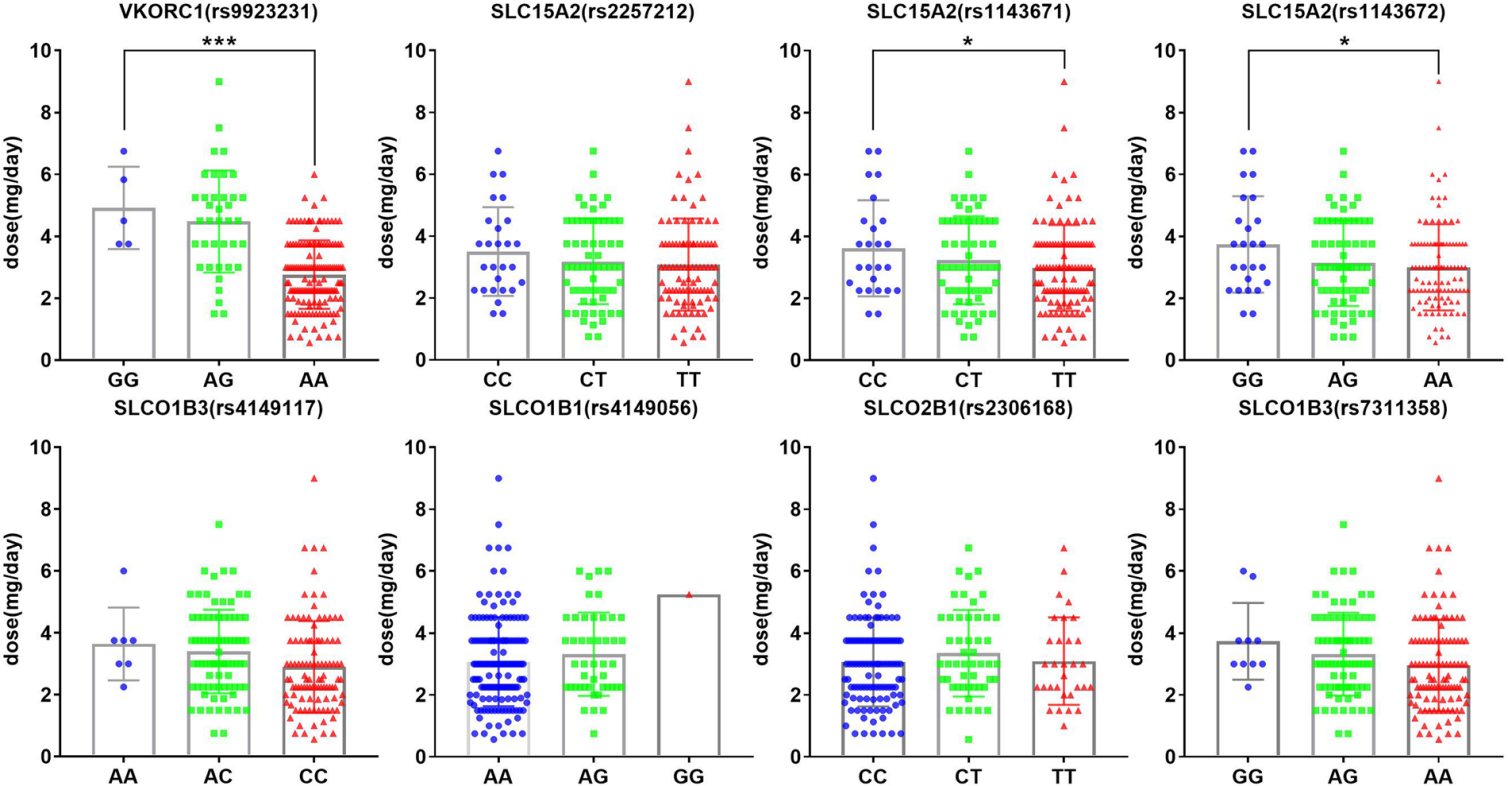
Relationships between stable warfarin dose and eight genetic polymorphisms. The effects of genetic polymorphisms on stable warfarin dose are shown as the differences in warfarin dose between every variant genotype (heterozygote or minor homozygote) and wild type. The distribution of warfarin doses is shown according to genotypes of SNPs. Data regarding warfarin dose are expressed as the mean ± SD. *Represents p < 0.05, **Represents p < 0.01, ***Represents p < 0.001 (analysed by a t test using GraphPad Prism Software version 5.0).

**Figure 3:**
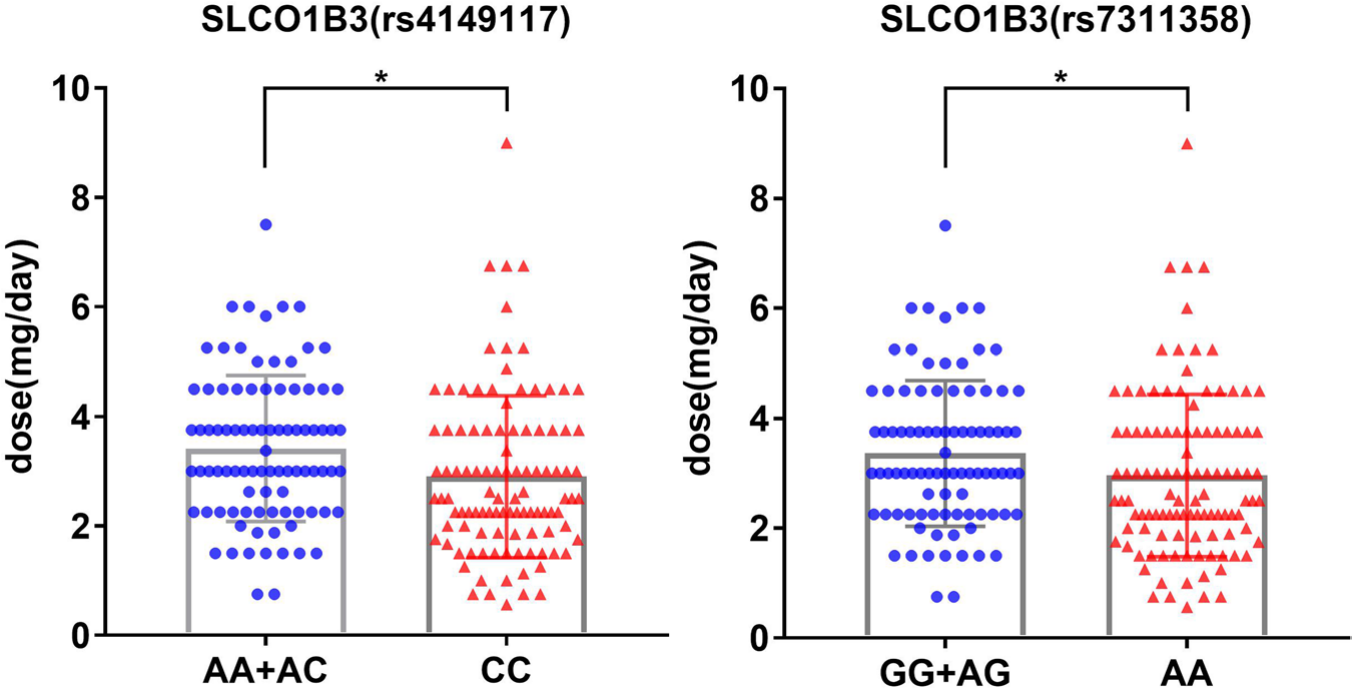
The association of *SLCO1B3* with warfarin maintenance dose. Data regarding warfarin dose are expressed as the mean ± SD. *Represents p < 0.05 (analysed by a T test using GraphPad Prism Software version 5.0).

**Figure 4:**
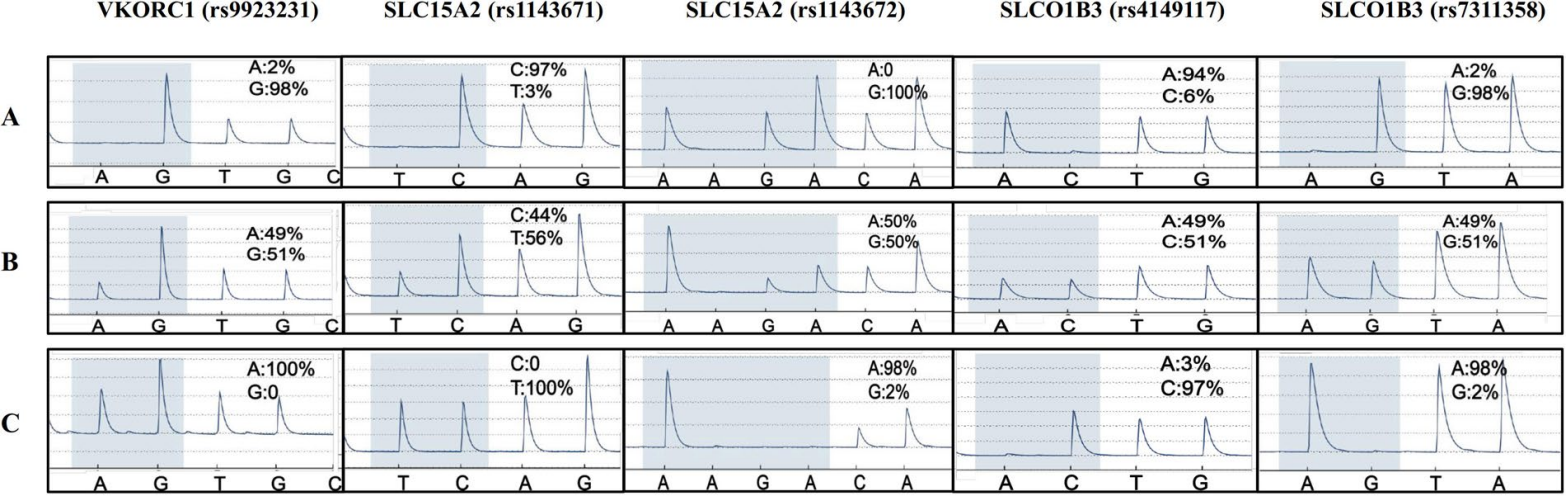
The results of pyrosequencing-based genotyping of *VKORC1*, *SLC15A2*, and *SLCO1B3* polymorphisms. A represents the major homozygote (wild type) of rs9923231 (*VKORC1*), rs1143671 (*SLC15A2*), rs1143672 (*SLC15A2*), rs4149117 (*SLCO1B3*), and rs7311358 (*SLCO1B3*). B represents the heterozygote of rs9923231 (*VKORC1*), rs1143671 (*SLC15A2*), rs1143672 (*SLC15A2*), rs4149117 (*SLCO1B3*), and rs7311358 (*SLCO1B3*). C represents the minor homozygote of rs9923231 (*VKORC1*), rs1143671 (*SLC15A2*), rs1143672 (*SLC15A2*), rs4149117 (*SLCO1B3*), and rs7311358 (*SLCO1B3*).

To more thoroughly explore the association of those SNPs with the warfarin maintenance dose, we performed quantitative data analysis on all of the SNPs using t tests and confirmed the significant associations of three SNPs, rs9923231 (p < 0.0001) in *VKORC1* and rs1143671 (p = 0.046) and rs1143672 (p = 0.022) in *SLC15A2*, with warfarin maintenance dose. As shown in Fig. 2, the mean daily warfarin dose for those subjects carrying the mutated genotype (AA for rs9923231 in *VKORC1*, TT for rs1143671 in *SLC15A2*, AA for rs1143672 in *SLC15A2*) was significantly lower than that for those carrying the wild type genotype (GG for rs9923231, CC for rs1143671, GG for rs1143672). They required dose values were 2.76 ± 0.09 mg/day for AA genotypes in rs9923231 (n = 149), 2.98 ± 0.13 mg/day for TT genotypes in rs1143671 (n = 111) and 3.00 ± 0.14 mg/day for AA genotypes in rs1143671 (n = 105). Those subjects with GG genotypes in rs9923231, CC genotypes in rs1143671 and GG genotypes in rs1143672 required mean daily doses of 4.92 ± 0.60 mg/day (n = 5), 3.62 ± 0.31 mg/day (n = 25) and 3.74 ± 0.31 mg/day (n = 25), respectively. Those subjects carrying the mutated genotypes of rs4149117 (CC) or rs7311358 (AA) did not have a significantly different maintenance dose of warfarin compared with that of subjects carrying the wild type genotype (AA for rs4149117, GG for rs7311358). However, both rs4149117 (p = 0.011) and rs7311358 (p = 0.046) showed a significant association with the warfarin maintenance dose difference between the wild type genotype and non-wild type genotype (Fig. 3). The mean daily dose for those subjects carrying the homozygous minor alleles (CC for rs4149117, AA for rs7311358) was significantly lower than that for subjects carrying 0 or 1 minor alleles (Fig. 3). The dose values were 2.90 ± 0.14 mg/day for CC genotypes in rs4149117 (n = 105) and 2.96 ± 0.14 mg/day for AA genotypes in rs7311358 (n = 110), while those subjects with AA or AC genotypes in rs9923231 required a mean daily dose of 3.42 ± 0.14 mg/day (n = 90). Those subjects with GG or AG genotypes in rs7311358 required a mean daily dose of 3.36 ± 0.14 mg/day (n = 89). Therefore, *VKORC1* (rs9923231), *SLC15A2* (rs1143671, rs1143672) and *SLCO1B3* (rs4149117, rs7311358) were strongly suggested to be important biomarkers for warfarin dose prediction. The results of pyrosequencing for those 5 polymorphisms are shown in Fig. 4. The other 3 SNPs, rs2257212 in *SLC15A2*, rs4149056 in *SLCO1B1* and rs2306168 in *SLCO2B1*, did not show any significant association with warfarin dose in the quantitative analysis. However, given their significant differences in the microarray screening analysis, those three SNPs must be further assessed in more Chinese patients.

## Discussion

The aims of the present study were to perform a large screening of risk genes and to decipher those variants that are likely to affect warfarin dosing in the Chinese population through ADME microarray analysis and pyrosequencing. We confirmed *VKORC1* as an important warfarin dose-related gene and identified two novel genes significantly associated with the maintenance dose of warfarin. Those novel variants may be considered as useful biomarkers for warfarin dose adjustment in clinical practice.

*VKORC1* and *CYP2C9* are the primary genes contributing to warfarin dose requirements^22,23^. They encode the vitamin K epoxide reductase complex 1 and cytochrome P450 2C9, respectively^22,23^. VKORC1 is the target protein for warfarin and inhibits the rate-limiting enzyme in the vitamin K regeneration cycle, thus preventing reduction of vitamin K to vitamin KH2, while the CYP2C9 enzyme metabolizes the more potent S-enantiomer of warfarin^22,23^. Multiple candidate gene studies and genome-wide association studies have consistently demonstrated that genetic polymorphisms in *VKORC1* and *CYP2C9* influence the inter-patient warfarin dose variation across various ethnic groups, together accounting for up to 50% of the total variability in warfarin dose requirements^7,24–26^. The Food and Drug Administration (FDA) has shown that SNPs in both genes, including *VKORC1*-1639G > A (rs9923231), CYP2C9*2 (rs1799853), and CYP2C9*3 (rs1057910), can be used as genetic variables to predict the required warfarin dose^26^. In accordance with those previous studies^13,14,24^, our results confirmed that *VKORC1* rs9923231 is the primary genetic determinant of variability in warfarin dose requirements in the Chinese population through use of a Chi-squared test and quantitative data analysis. However, the common variants in *CYP2C9* were not found to be significantly associated with warfarin dose requirements in the present study. This failure in the identification of significant *CYP2C9* variants (rs1799853 or rs1057910) was likely due to the low frequency of the minor alleles (MAF: 0.0048 for rs1799853, MAF: 0.0476 for rs1057910) in the Southern Han Chinese population (see https://www.ncbi.nlm.nih.gov/variation/tools/1000genomes/). Our results are also supported by many previous studies in China, although those studies revealed significant associations of CYP2C9*2 and CYP2C9*3 with warfarin dosing^23,27^. In addition, associations of *CYP2C9* variants with warfarin dose may also be masked by the strong contribution of *VKORC1* rs9923231 to warfarin dosing variability.

The most interesting finding of this study was the identification of the two most common variations (rs1143671 and rs1143672) in *SLC15A2* as important predictors of the warfarin maintenance dose. *SLC15A2* encodes the H+/peptide transporter 2 (PEPT2), which belongs to the solute carrier 15 family (SLC15)^28^. It is a high-affinity and low-capacity type proton-coupled oligopeptide transporter that is widely expressed in the kidney, lung, eye, prostate and mammary gland and in the central nervous system^28^. As with PEPT1, PEPT2 mediates proton-coupled active transport of a broad range of dipeptides and tripeptides as well as a variety of peptide-like drugs (such as β-lactam antibiotics, cefadroxil, penicillins, and angiotensin-converting enzyme (ACE) inhibitors)^28,29^. Genetic variants in human PEPT2 may lead to functional changes in transport, thereby potentially affecting the renal clearance of PEPT2 drug substrates and influencing drug pharmacokinetics and toxicities^28,30^. There are two main variants of PEPT2 that differ in three amino acid positions, PEPT2*1 (containing amino acids L350, P409, and R509) and PEPT2*2 (containing amino acids F350, S409, and K509)^28,30,31^. PEPT2*1 is evenly distributed among Caucasians and Africans, while PEPT2*2 exists at higher frequencies in Asians^31,32^. Compared with the data from the HapMap database, the frequencies of rs2257212 (L350F), rs1143671 (P409S) and rs1143672 (R509K) were higher in the present study, with values of 47.8%, 55.2% and 52.2%, respectively (Table 5). Furthermore, our ADME microarray analysis revealed that three non-synonymous single nucleotide variations in this gene, rs2257212 (L350F), rs1143671 (P409S) and rs1143672 (R509K) were significantly associated with warfarin dosing. Unlike rs2257212 (L350F), both rs1143671 (P409S) and rs1143672 (R509K) were further validated by the subsequent pyrosequencing study. As for rs9923231 in *VKORC1*, both SNPs in *SLC15A2*, rs1143671 and rs1143672, were also shown to be significantly associated with warfarin dose in the dominant model of the quantitative data analysis. Furthermore, carriers of rs1143671 or rs1143672 should be given significantly lower warfarin doses than those subjects with a wild type genotype. The reduced warfarin dose requirement in subjects with those *SLC15A2* variants may result from changes in the response of PEPT2 to this drug. The variant rs1920305 (R57H) of PEPT2 was shown to cause a complete loss of transport function^33^. This hypothesis is also supported by previous studies that found that both variants in *SLC15A2* (rs1143671 and rs1143672) had 3-fold higher km values for glycylsarcosine (Gly-Sar) and displayed different pH sensitivities for Gly-Sar uptake compared to the wild type genotype^31^. The effects of rs1143671 and rs1143672 on the transport function of PEPT2 and the pharmacodynamics and pharmacokinetics of warfarin remain unclear to date. It is necessary that future studies explore the functional activity of those variants in the transport of warfarin. Here, we believe that subjects with those genetic variations may have altered responses to warfarin and therefore must be given a lower warfarin dose.

Another novel finding from our study was that, as with *VKORC1* and *SLC15A2*, the two most common variations (rs4149117 and rs7311358) in *SLCO1B3* were also suggested to be important predictors of the warfarin maintenance dose. *SLCO1B3* encodes the organic anion transporting polypeptide 1B3 (OATP1B3), which is abundantly expressed in the perivenous section of the liver and mediates the hepatic uptake of endogenous compounds (such as bile acids, dehydroepiandrosterone-3-sulfate, peptide hormones such as cholecystokinin-8 (CCK-8)) and drugs such as lipid-lowering statins (rosuvastatin or atorvastatin) and anti-cancer agents (irinotecan or methotrexate), thereby influencing hepatobiliary elimination^34–36^. For *SLCO1B3*, several single-nucleotide polymorphisms have been identified in coding and non-coding regions of the gene to date. At least three non-synonymous SNPs, including rs4149117 (*SLCO1B3*-c.334 T > C), encoding OATP1B3-p.S112A, rs7311358 (*SLCO1B3*-c.699 G > A), encoding OATP1B3-p.M233I, and rs72559743 (*SLCO1B3*-c.1564 G > T), encoding OATP1B3-p.G522C, have been found to increase the transporter activity *in vitro* to date^37,38^. In the present study, we for the first time found that rs4149117 (S112A) and rs7311358 (M233I) in *SLCO1B3* were significantly associated with warfarin dose in the recessive model as assessed with a quantitative data analysis, unlike rs1143671 and rs1143672 in *SLC15A2*. Those subjects with homozygous minor alleles in rs4149117 or rs7311358 were suggested to be given a lower warfarin dose than those carrying the wild type allele. Frymoyer *et al*. found that warfarin may be a substrate of OATP1B3, although a change in the pharmacokinetics (PK) of warfarin was not observed in the presence of an OATP1B3 inhibitor^39^. Whether both non-synonymous SNPs rs4149117 (S112A) and rs7311358 (M233I) could alter the PK of warfarin remains unclear. However, previous studies revealed that both rs4149117 (S112A) and rs7311358 (M233I) could influence the pharmacokinetic profile of another substrate, the glucuronide metabolite (MPAG) of the immunosuppressant mycophenolic acid (MPA)^40,41^. In addition, four functional variations including rs12299012 (V560A), rs559692629 (H520P), rs7311358 (M233I), and rs4149117 (S112A) were recently shown to decrease uptake activity compared to that of the wild type gene for rosuvastatin and cholecystokinin-8 (CCK8) in HeLa cells^42^. This evidence implied the clinical relevance of these two variants in the PK of warfarin. Thus, we thought that the variability of warfarin dosing in carriers with rs4149117and rs7311358 may be attributed to the decreased response of variants to this drug and that the ensuing change in the pharmacokinetics of warfarin is the putative effect of rs1143671 and rs1143672 on warfarin dosing.

Unfortunately, unlike rs2257212 in *SLC15A2*, both rs4149056 in *SLCO1B1* and rs2306168 in *SLCO2B1* were identified as significant variants associated with warfarin maintenance dose in the ADME microarray analysis but could not be validated in the pyrosequencing data-based qualitative and quantitative analyses. *SLCO1B1* and *SLCO2B1* encode OATP1B1 and OATO2B1, respectively, which belong to members of the organic anion transporting polypeptide (OATP) family^34–36,43^. As with OATP1B3, both OATP1B1 and OATO2B1 are major uptake transporters on the sinusoidal membrane of human hepatocytes^34–36,43^. It was found that the common polymorphism rs4149056 (*SLCO1B1*-c.521 T > C), encoding OATP1B1-p.V174A, could down-regulate the expression of OATP1B1 protein and consequently decrease its transport activity, and this polymorphism could contribute to the high variability in the pharmacokinetics and pharmacodynamics of raloxifene between individuals, as with another variation, rs2306283 (*SLCO1B1*-c.388 A > G), encoding OATP1B1-p.N130D^44–46^. Additionally, it has been reported that the rs4149056 (V174A) variant of SLCO1B1 is associated with changes in the transporter activities for pravastatin, 17b-D-glucuronide and estrone sulfate *in vitro* ^44,47–49^. Moreover, the CC genotype of rs4149056 has been associated with increased plasma concentrations of statins and glinides in clinical studies^50–52^. The common polymorphism rs2306168 (*SLCO2B1*-c.1457 C > T), encoding OATP2B1-p.S486F, was shown to reduce uptake of atorvastatin, rosuvastatin and the prototypical substrate estrone sulfate^46^. A recent study revealed that rs2306168 (S486F) could change the pharmacokinetics of celiprolol, leading to lower celiprolol concentrations in individuals with the rs2306168 (S486F) homozygous genotype than in those with other genotypes following administration of the therapeutic dose of a test drug^53^. Given the importance of rs4149056 (V174A) in *SLCO1B1* and rs2306168 (S486F) in *SLCO2B1* for transporter activity, the association of both SNPs with warfarin dose and their effects on the pharmacokinetics and pharmacodynamics of this drug must be investigated in other cohorts with large sample sizes and in animal models, respectively, in the future.

The limitations of the study are as follows: the small sample size decreases the available statistical power to detect potentially associated SNPs; the validation of those SNPs identified from ADME microarray analysis was not done in an independent Cohort of completely different patients but in a pooled cohort including microarray screening samples and limited replication samples.

## Conclusion

In the present study, we for the first time identified two novel genes, *SLC15A2* and *SLCO1B3*, and found that four common functional polymorphisms in those two genes, rs1143671 and rs1143672 in *SLC15A2* and rs4149117 and rs7311358 in *SLCO1B3*, were significantly associated with the warfarin maintenance dose in the Han-Chinese population using high throughput ADME microarray screening and pyrosequencing technologies. The identified variants were suggested to be efficient predictors for the variability of the warfarin maintenance dose. More large well-designed clinical trials are needed to further confirm and define the optimal approach for using these pharmacogenetic factors for warfarin dose prediction in clinical practice.

## Materials and Methods

### Study subjects and study design

Patients with cardiac valve replacement or atrial fibrillation who met the following criteria were eligible for inclusion in the study: 1) part of the Han Chinese population, 2) more than 18 years old, 3) no hepatic or renal diseases, 4) would sign written informed consent and finish questionnaires, and 5) no evidence or history of haemorrhage or thrombosis complications during the warfarin therapy. A total of 156 patients were first enrolled as the “screening cohort” from 2012 to 2014. An additional 57 patients were enrolled as the “replication cohort” in 2015. Together with those subjects from the “screening cohort”, those 57 subjects were included in the subsequent pyrosequencing. Finally, a total of 201 patients were enrolled in this study. The study was approved by the Ethics Committee of Zhongshan Hospital, Xiamen University, China, and it was conducted according to the declaration of Helsinki. The study design is shown in Fig. 1A.

All study participants were on stabilized anticoagulation with warfarin for prevention of thromboembolism. The mean daily maintenance dose (mg/day) of warfarin was defined as “patients receiving the same dose of warfarin for a period of 3 months with three or more consecutive international normalized ratio (INR) measurements in a 1-week interval within target range (2.0–3.5)”. The enrolled patients were then classified into “high dose”, “medium dose”, and “low dose” groups according to the mean daily maintenance dose of warfarin.

Warfarin doses ≥4 mg/day and ≤2 mg/day were considered as high dose and low dose, respectively, while the medium dose varied from 2 mg/day to 4 mg/day (2 mg/day < medium dose <4 mg/day). Dose modifications were made based on American College of Chest Physician (ACCP) guidelines (8th edition) for warfarin therapy. Data on each patient’s age, height, weight, body mass index, medication history, INR values, warfarin dose and living habits were obtained from the patient’s case records and a follow-up telephone call.

Blood specimens from all the 201 study patients were separated by centrifugation at 4000 rpm for 10 minutes to get the plasma in the commercial evacuated tubes containing 3.2% sodium citrate. Then, the time analysis was performed using a STAGO STA-R automatic laboratory coagulometer (Diagnostica Stago S.A.S, Asnières sur Seine, France) and a STA-Neoplastine CI Plus kit (Diagnostica Stago S.A.S, Asnières sur Seine, France), and INR values were calculated subsequently.

### Genotyping by VeraCode ADME Core Panel and data analysis

For each locus, there is a biotinylated oligonucleotide that copies a specific genomic region. Genotype determination is then accomplished by performing the allele-specific extension and ligation on the copied region. Verascan software was used to make genotype calls and generate quality control metrics, including call rates, average signal, cluster quality, and reproducibility. To ensure quality control and minimize false-positive and false-negative data, the VeraCode ADME Core Panel includes multiple types of internal controls in each sample, such as a sample tracking control, process control, subpool position control, hybridization control, and assay control. In the genotyping data analysis, the SNP call rate threshold for the SNPs in the total samples was set at 95% in practice. Haploview software (Broad Institute, Cambridge, MA) was used to generate linkage disequilibrium estimates.

### Pyrosequencing assay for genotyping data

DNA was extracted from whole blood using MagCore Nucleic Acid Extraction Kits with a MagCore HF48 Automated Nucleic Acid Extractor (RBC BIOSCIENCE, China) and was then used as the template for PCR amplification. The 8 significant SNPs identified in the ADME microarray analysis were validated through pyrosequencing. All of the primers for PCR and sequencing were designed by PyroMark Assay Design 2.0 Software.

### Statistical analysis

Demographic data are expressed as the mean ± standard deviation (SD) for continuous variables and as numbers (percentages) for categorical variables. The differences in allele frequency and genotype frequency for the 8 significant single nucleotide polymorphisms (SNPs) included in the pyrosequencing study among the three groups (high dose, medium dose, low dose) were assessed using the Pearson’s chi-squared test. Those SNPs included rs9923231 in *VKORC1*, rs2306168 and rs2257212 in *SLCO2B1*, rs1143671 and rs1143672 in *SLC15A2*, rs4149056 in *SLCO1B1* and rs4149117 and rs7311358 in *SLCO1B3*. We used 4 genetic models, the general genetic model, the dominant model, the recessive model, and the multiplicative model (allele test) to analyse the influence of each SNP on warfarin dose classification (high, medium and low). The minor allele and the major allele were defined according to the frequency of the allele in Americans. The major allele and major homozygotes were used as the references in the allele test and the genotype test, respectively. To exclude or reduce type I errors in the association analysis of each SNP, Bonferroni corrections were used to adjust the p value for multiple testing in the χ^2^ test–based qualitative analysis. To adjust for the effect of clinical covariates including age, gender, height and weight and to prevent any spurious associations, we analysed the association of every SNP with warfarin dose using an ordinal logistic regression method. Additionally, we performed a quantitative analysis to determine the association of the genotypes with warfarin dose using t tests. A two-tailed p value < 0.05 was considered statistically significant in all genetics tests. All statistical analyses were performed using SPSS 17.0 software (SPSS Inc., Chicago, IL) and GraphPad Prism Software version 5.0 (GraphPad, USA).
